# Clinical features of invasive bronchial-pulmonary aspergillosis in critically ill patients with chronic obstructive respiratory diseases: a prospective study

**DOI:** 10.1186/cc9402

**Published:** 2011-01-06

**Authors:** Hangyong He, Lin Ding, Fang Li, Qingyuan Zhan

**Affiliations:** 1 Department of Respiratory and Critical Care Medicine, Beijing Institute of Respiratory Medicine, Beijing Chao-Yang Hospital, Capital Medical University, 8 Gongren Tiyuchang South Road, Beijing, 100020, PR China; 2 Department of Infectious Diseases and Clinical Microbiology, Beijing Institute of Respiratory Medicine, Beijing Chao-Yang Hospital, Capital Medical University, 8 Gongren Tiyuchang South Road, Beijing, 100020, PR China

## Abstract

**Introduction:**

Critically ill patients with chronic obstructive respiratory diseases (CORD) who require intensive care unit (ICU) admission are at particular risk for invasive bronchial-pulmonary aspergillosis (IBPA). The purpose of this study is to investigate clinical features for rapid recognition of IBPA in critically ill patients with CORD.

**Methods:**

We included 55 consecutive CORD patients in a respiratory ICU in a prospective, single-center, cohort study. In this study, IBPA combined two entities: ATB and IPA.

**Results:**

Thirteen of 55 patients were diagnosed with IBPA. Before ICU admission, three variables were independent predictors of IBPA with statistical significance: more than three kinds of antibiotics used before the ICU admission, accumulated doses of corticosteroids (>350 mg) received before the ICU admission, and APACHE II scores >18 (OR, 1.208; *P *= 0.022; OR, 8.661; *P *= 0.038; and OR, 19.488; *P *= 0.008, respectively). After ICU admission, more IBPA patients had a high fever (>38.5°C) (46.2% versus 11.9%; *P *= 0.021), wheeze without exertion (84.6% versus 50.0%; *P *= 0.027), dry rales (84.6% versus 40.4%; *P *= 0.005), higher white blood cell counts (21 × 10^9^/L versus 9.4 × 10^9^/L; *P *= 0.012), lower mean arterial pressures (77.9 mm Hg versus 90.5 mm Hg; *P *= 0.019), and serum creatinine clearances (36.2 ml/min versus 68.8 ml/min; *P *< 0.001), and liver-function and coagulation abnormalities. Bronchospasm, sputum ropiness, and plaque formation were more common for IBPA patients during bronchoscopy (66.7% versus 14.3%; *P *= 0.082; 18% versus 0; *P *= 0.169; and 73% versus 13%; *P *= 0.003, respectively). More IBPA patients had nodules and patchiness on chest radiograph on day 1 of admission, which rapidly progressed to consolidation on day 7. IBPA mortality was higher than that of non-IBPA patients (69.2% versus 16.7%; *P *= 0.001).

**Conclusions:**

IBPA may be suspected in critically ill CORD patients with respiratory failure and clinical and bronchoscopic manifestations of severe infection, bronchospasm, and rapid progression of radiologic lesions that are irresponsive to steroids and antibiotics. To avoid misdiagnosis and establish the microbiologic etiology, early bronchoscopy and tight radiologic follow-up should be performed.

## Introduction

*Aspergillus *tracheobronchitis (ATB) and invasive pulmonary aspergillosis (IPA) are two clinical presentations of invasive aspergillosis (IA) [1]. The predisposing factors for ATB and IPA are similar [1,2]. Neutropenic and immunocompromised patients are particularly at risk. Chronic obstructive respiratory disease (CORD) is defined as chronic obstructive diseases of the airways and pulmonary tissues. CORD includes a wide array of serious diseases, and chronic obstructive pulmonary disease (COPD), bronchial asthma, and bronchiectasis are common CORDs [3]. Patients with CORD frequently experience acute exacerbations of their underlying illnesses that require hospitalization or intensive care unit (ICU) admission. Recent reports suggest that the incidence of IA appears to be increasing in CORD patients requiring ICU admission [4-6]. Moreover, CORD was a major component of critically ill patients with IA in the ICU. Despite invasive ventilation and antifungal treatments, the mortality due to IPA for critically ill COPD patients remains at 67% to 100% [2,6-10], and the mortality for ATB is 80% [2].

These high mortality rates may be the result of unclear clinical features and the delayed diagnoses and treatments for ATB and IPA among CORD patients. Recently, Tasci *et al*. [2] described the clinical features of ATB and proposed an optimal diagnostic strategy. Bulpa *et al*. [7] proposed a series of diagnostic criteria for IPA in the COPD population, which provided the criteria for the clinical diagnosis of IPA and ATB. However, these results were based on retrospective studies [2,7].

Several reports have suggested that ATB might progress to or coexist with IPA [11,12]. A recent study showed that ATB could occur in moderately or non-immunocompromised patients with impaired airway structures or defense functions and may be an early stage of IPA [13]. ATB and IPA might be two phases or manifestations of one entity, invasive bronchial-pulmonary aspergillosis (IBPA), which was rarely recognized before. In the present study, we preferred to combine IPA and ATB as one disease, and we used the term IBPA to indicate these two subentities.

The aim of this single-center prospective cohort study was to describe the early clinical signs and to evaluate the available diagnostic procedures for IBPA in critically ill CORD patients in our ICU to assess their importance for rapid recognition and appropriate treatment.

## Materials and methods

### Study population and data collection

In our study, all of the patients were admitted to an ICU because of respiratory failure from February 2007 to November 2008. These patients were older than 18 years and had been diagnosed with either severe COPD, stage III or IV according to the Global Initiative for Chronic Obstructive Lung Disease (GOLD), moderate or severe persistent bronchial asthma according to Global Initiative for Asthma (GINA), or bronchiectasis with respiratory failure according to their clinical history, symptoms, signs, and laboratory findings.

The following information was stored in a data file: patients' characteristics, including age, sex, medical history, and reasons for ICU admission; use of immunosuppressive drugs (steroids and others) and antibiotics; presence of typical symptoms and signs; and standard ICU laboratory findings on days 1, 4, and 7 after admission, including complete blood count, arterial blood gas analysis (ABGA), serum biochemistry tests, activated partial thromboplastin time (APTT), and microbiologic examination; and disease severity, assessed according to the Acute Physiology and Chronic Health Evaluation II (APACHE II) on their admission to the ICU.

A sandwich enzyme-linked immunosorbent assay (ELISA) for galactomannan (GM) detection (Platelia *Aspergillus*; Sanofi Diagnostics Pasteur, Marnes-La-Coquette, France) was used according to the manufacturer's instructions. Serum sampling for GM detection was done on days 1, 4, and 7 after ICU admission. An optical density (OD) ratio of 0.5 or greater for GM in serum was considered positive.

Fiberoptic bronchoscopy with bronchoalveolar lavage was performed on days 1, 4, and 7 of inclusion if the patient was intubated and if feasible. The selection of sampling areas was based on the infiltrate location on a chest radiograph. The presence of any tracheal or bronchial lesions was recorded by the endoscopist (QZ). Lavage samples were submitted for direct microscopic examination and bacterial and fungal cultures.

A chest radiograph (CXR) by bedside was done on days 1, 4, and 7 after ICU admission. Pulmonary computed tomography (CT) was also done if feasible, depending on the patients' situations.

Antifungal treatment was started and selected at the discretion of the attending physician (QZ) and was not protocol defined.

The study was approved by the ethics committee, and written, informed consents were obtained from the patients or their next of kin.

### Processing of clinical samples

LRT samples of all patients included in this study were taken once a day for the first 3 days of their ICU stays. LRT samples were collected again once per week if the patient remained in the ICU for more than 7 days. All LRT specimens were cultured on conventional media, including sheep-blood agar and chocolate agar. At the same time, all LRT specimens were cultured on CHROMagar medium and Sabouraud dextrose agar. Cultures were incubated at 25°C and 37°C, respectively, for 7 to 14 days. When spore growth was suboptimal on the routine media, LRT samples were further cultured on potato dextrose agar for a better conidial production. *Aspergillus *isolates were identified by using standard morphologic procedures, including colonial morphology, growth velocity, color, morphology of hyphae, and characteristics of hyphae and spores under microscopy.

### Case definitions of IBPA

According to the definitions of invasive fungal disease of the European Organization for Research and Treatment of Cancer/Mycoses Study Group (EORTC/MSG), ATB is diagnosed when tracheobronchial ulceration, nodule, pseudomembrane, plaque, or eschar is seen on bronchoscopic analysis, which is confirmed by biopsy or positive culture for *Aspergillus *or both [1].

IPA was classified as "proven," "probable," or "possible," based on case definitions of EORTC/MSG [1]. Proven IPA referred to histopathologic evidence of tissue invasion by septated, acutely branching filamentous fungi together with a positive culture. Probable IPA referred to the presence of a positive culture or cytology for *Aspergillus *species from any lower respiratory tract (LRT) sample together with one major criterion (halo sign, air-crescent sign, or cavity within an area of consolidation on CT scan) or two of three minor clinical criteria (symptoms of LRT infection, pleural rub, or new infiltrate without an alternative diagnosis). Possible infection referred to patients who fulfilled probable infection criteria but did not have a positive *Aspergillus *culture or microscopy from LRT, or serology. Patients with positive cultures for *Aspergillus *from nonsterile sites, but without any other evidence of fungal infection, were considered to be colonized. Diagnosis was not based on a serum GM test.

The diagnosis of IBPA referred to a patient diagnosed with ATB or IPA or both. Patients diagnosed with ATB or IPA or both were included in the IBPA group. Among critically ill patients with COPD, bronchial asthma, and bronchiectasis, those diagnosed as non-IBPA were included in the non-IBPA group. Patients with *Aspergillus *colonization were considered noninfected cases and were included in the non-IBPA group.

### Statistical analysis

Patients with CORD admitted to the ICU were divided into IBPA and non-IBPA groups. The clinical signs and results of diagnostic tests were compared between the two groups. Normally distributed continuous variables were expressed as mean ± SD and compared with a *t *test. Non-normally distributed continuous variables were expressed as median and quartiles and compared with the Wilcoxon rank-sum test. Categoric variables were compared with a χ^2 ^test. Multivariate logistic-regression analysis was used to identify independent risk factors for IBPA patients. The *P *values < 0.05 were considered significant. All analyses were carried out with the use of SPSS software for Windows (release 11.5).

## Results

### Patient characteristics

From February 2007 to November 2008, in total, 343 patients were admitted to our ICU. Fifty-five of these patients who met the inclusion criteria were enrolled: 47 (86%) had COPD, four (7%) had asthma, and four (7%) had bronchiectasis. The characteristics of the total study group are shown in Table 1.

**Table 1:** Patient characteristics

	IBPA	Control	*P *value
Number of patients	13	42	----
Demographic characteristics			
Age, mean, years (SD)	74.3 (13.5)	73.2 (7.46)	0.150
Sex, number (%)			
Male	7 (53.8)	25 (59.5)	0.228
Female	6 (46.2)	17 (40.5)	
Length of hospitalization before ICU admission, days (IQR)	15 (8.5-29.5)	3 (2-6.25)	0.001^a^
Length of ICU stay, days (IQR)	10 (6-20)	7 (5-14)	0.253
Transferred from other hospital/ICU, number (%)	5 (38.5)	5 (11.9)	0.079
From other ICU, number (%)	1 (7.7)	3 (7.1)	0.672
From other hospital, number (%)	4 (30.8)	2 (4.8)	0.034^a^
Medical history, number (%)			
Three or more hospitalizations	9 (69.2)	38 (90.5)	0.147
Chronic renal dysfunction	3 (23.1)	4 (9.5)	0.421
Diabetes mellitus	0 (0)	6 (14.3)	0.350
Nonhematologic malignancy	0 (0)	3 (7.1)	0.438
Corticosteroids use			
Number of patients with steroids use (%)	9 (69.2)	26 (61.9)	0.881
Prolonged steroids for≥3 weeks before ICU admission, number (%)	1 (7.7)	5 (11.9)	1.000
Accumulated dosage of systemic steroids,▵ mg, mean (SD)	371 (199)	180 (150)	0.006^a^
Accumulated dosage of systemic steroids,▵ mg, median (IQR)	400 (190-535)	105 (75-241)	0.021^a^
Duration of steroids before ICU admission, day, median (IQR)	6 (0-7)	1 (0-3)	0.041^a^
Daily dosage of systemic steroids ▵, mg, median (IQR)	50 (0-75)	50 (0-63)	0.377
Inhaled steroids, number (%)	1 (7.7)	2 (4.8)	1.000
Antibiotics			
Number of patients with antibiotics (%)	13 (100)	36 (85.7)	0.350
Number of kinds of antibiotics, median (IQR)	3 (1-5)	2 (1-2)	0.037^a^
Length of antibiotics use, days, median (IQR)	10 (4.5-22.0)	3 (2-10.3)	0.015^a^
APACHE II scores, mean (SD)	18.6 (7.1)	12.6 (4.5)	0.010^a^
Mechanical ventilation			
Total number of patients with invasive ventilation during RICU stays (%)	13 (100)	25 (59.5)	0.016^a^
Duration of invasive mechanical ventilation, days, median (IQR)	8 (5-15)	3 (0-10)	0.006^a^
Outcome, number (%)			
Survival	4 (30.8)	35 (83.3)	0.001^a^
Dead	9 (69.2)	7 (16.7)	

SD, standard deviation; IQR, interquartile range; APACHE II, Acute Physiology and Chronic Health Evaluation II. ▵The steroid doses were converted to prednisone dose (for example, 20 mg of hydrocortisone = approximately 5 mg of prednisone). ^a^*P *< 0.05.

Thirteen (24%) patients were diagnosed with IBPA, and the remaining patients (42) did not have IBPA. In IBPA group, 11 patients had COPD, one patient had asthma, and one patient had bronchiectasis. According to the diagnostic criteria for IBPA, the 13 IBPA patients were classified as proven (*n *= 4), probable (*n *= 8), and possible IBPA (*n *= 1) (Table 2). One case was diagnosed as colonized. *Aspergillus *spp. was the only mold pathogen, and no other non-*Aspergillus *invasive mold infection was found in the patients studied. IBPA cases were diagnosed at a median of 2 days (IQR, 1 to 7 days) after the patients' admission to the ICU. The reasons for respiratory failure in IBPA patients were infection (12 cases) and heart dysfunction (one case), which caused exacerbations of their underlying respiratory diseases. Eleven of the 13 IBPA patients had a positive culture or microscopic examination of *Aspergillus *spp. for their LRT samples collected at the first day of their ICU admission, and two patients had positive microbiologic results for LRT samples collected at day 6 and 8 after the ICU admission. As a result, these 11 cases were determined as having developed the infection before the ICU admission. All IBPA patients and 25 non-IBPA patients received invasive mechanical ventilation (100% versus 59.5%; *P *= 0.016). The duration of invasive mechanical ventilation for IBPA was significantly longer than that for non-IBPA patients (8 days versus 3 days; *P *= 0.006). The mortality for IBPA was higher than that of control group (69.2% versus 16.7%; *P *= 0.001). The causes of death for these nine IBPA patients were multiple organ failure for four cases, acute renal failure for three cases, and septic shock for two cases.

**Table 2:** Diagnosis, antifungal therapy, and patient outcomes in IBPA group

	Class of diagnosis	Type	Risk factors	Chest CT scan	Chest x-ray	BSP	Serum IgE (KU/L)	Asp Ab	Fungal culture	Aspergillus species	Histology	Previous antifungal prophylaxis	Antifungal therapy	**GM detection 0.5 ng/ml**^**a**^
1	Probable	IPA	Steroids and antibiotics	Nodule, patching	Patching, consolidation		513	Positive	ETA (1)	*A. fumigatus*		No	ABLC	Negative
2	Proven	ATB and IPA	Steroids and antibiotics	Nodule, consolidation, halo sign	Patching, nodule	PM	33	Negative	ETA (2); BALF (5)	*A. fumigatus*	Positive	No	ABLC and voriconazole	Positive
3	Probable	IPA	Steroids and antibiotics	Nodule, patching, consolidation	Patching		209	Positive	Spu (5)	*A. fumigatus*		Yes	ABLC and voriconazole	Positive
4	Probable	IPA	Steroids and antibiotics		Negative		51	Negative	Spu (2)	*A. fumigatus*		No	ABLC and itraconazole	Negative
5	Proven	ATB	Steroids and antibiotics		Negative	PM	148	Positive	ETA (9); BALF (1)	*A. fumigatus and A. flavus*	Positive	No	ABLC	Positive
6	Probable	IPA	Steroids and antibiotics		Patching, nodule, consolidation		14	Negative	ETA (12)	*A. fumigatus*		No	ABLC and voriconazole	Positive
7	Possible	IPA	Antibiotics	Patching, nodule, cavitation	Patching, nodule, consolidation, cavitation		15	Negative	No	No		No	Caspofugin and voriconazole	Negative
8	Probable	IPA	Steroids and antibiotics		Patching		4	Negative	ETA (2)	*A. fumigatus*		No	No	Positive
9	Probable	IPA	Steroids and antibiotics		Nodule, consolidation		67	Negative	Spu (3)	*A. flavus*		No	ABLC	Negative
10	Probable	IPA	Steroids and antibiotics	Patching, nodule, consolidation	Patching, nodule, consolidation		29	Negative	Spu (3)	*A. niger*		No	ABLC and voriconazole	Positive
11	Proven	ATB and IPA	Antibiotics	Nodule, consolidation	Patching, nodule, consolidation	PM	89	Positive	Spu (3)	*A. niger*	Positive	Yes	ABLC and caspofugin	Negative
12	Proven	ATB and IPA	Steroids and antibiotics		Patching	PM	107	Negative	BALF (5)	*A. fumigatus*	Positive	No	ABLC	Positive
13	Probable	IPA	Antibiotics		Patching, consolidation		260	Positive	ETA (2)	*A. fumigatus*		No	ABLC	Negative

ABLC, amphotericin B lipid complex; Asp Ab, serum *Aspergillus *antibody; ATB, *Aspergillus *tracheobronchitis; BALF, bronchoalveolar lavage fluid; BSP, bronchoscopy; ETA, endotracheal aspiration; GM, galactomannan; IgE, immunoglobulin E; IPA, invasive pulmonary aspergillosis; PM, pseudomembrane; Spu, sputum. "number" in the fungal-culture column, times of positive cultures. ^**a**^Positive means one positive in any of the three samples.

Four patients had tracheobronchial mucus biopsies, and two of them had lung biopsies; no autopsy was obtained for this study.

In the IBPA group, 13 patients had a length of ICU stay of more than 1 day, 12 patients for more than 4 days, and 9 patients for more than 7 days, respectively. In the non-IBPA patients, 42 cases, 36 cases, and 23 cases stayed in the ICU for more than 1, 4, and 7 days, respectively.

### Steroids and antibiotics

#### Steroids

The dosages of systemic steroids received by all patients were converted to prednisone or equivalent doses by steroid potency (for example, 20 mg of hydrocortisone = 5 mg of prednisone). The numbers of patients who received steroids treatment before ICU admission in the IBPA and non-IBPA groups were similar (69% versus 62%). Compared with non-IBPA patients, before their admissions to the ICU, IBPA patients received a significantly higher mean dosage of systemic steroids (371 mg versus 180 mg of prednisone or equivalent; *P *= 0.006). IBPA patients received steroids for a longer period than did non-IBPA patients (median, 6 days versus 1 day). The median daily dosages of systemic steroids received by IBPA and non-IBPA patients were similar (Table 1).

#### Antibiotics

Most patients in the two groups received antibiotics treatment before their ICU admissions. The IBPA patients were given significantly more kinds of antibiotics for a longer treatment period than were the non-IBPA patients (Table 1).

### Symptoms and Signs

#### Symptoms

More IBPA patients had high fevers did non-IBPA patients (T >38°C; 46% versus 12%; *P *= 0.021). Compared with non-IBPA patients, wheeze without exertion was a more common symptom for IBPA patients (85% versus 50%). Hemoptysis and chest pain were rare in both groups (Table 3).

**Table 3:** Clinical characteristics (symptoms and signs)

	IBPA	Control	*P *value
Number of patients	13	42	----
Symptoms, number (%)			
Fever	8 (61.5)	10 (23.8)	0.028^a^
Body temperature >38.5°C	6 (46.2)	5 (11.9)	0.021^a^
Cough	10 (76.9)	36 (85.7)	0.749
Wheeze	11 (84.6)	36 (85.7)	1.000
Wheeze with exertion	0 (0)	15 (35.7)	0.030^a^
Wheeze without exertion	11 (84.6)	21 (50)	0.027^a^
Sputum production	10 (76.9)	37 (88.1)	0.583
Phlegm	3 (23.1)	5 (11.9)	0.583
Hemoptysis	2 (15.4)	1 (2.4)	0.136
Chest pain	2 (15.4)	2 (4.8)	0.234
Signs at ICU admission			
Body temperature, °C, mean (SD)	36.6 (0.5)	36.7 (0.6)	0.876
Heart rate, beats per minute, mean (SD)	106.5 (23.9)	95.2 (21.2)	0.108
Respiratory rate, breaths per minute, mean (SD)	28.3 (8.5)	24.3 (11.4)	0.243
Mean arterial pressure, mean (SD)	77.9 (14.2)	90.5 (17.0)	0.019^a^
Rales, number (%)			
Dry rales	11 (84.6)	17 (40.4)	0.005^a^
Moist rales	9 (69.2)	30 (71.4)	1.000

SD, standard deviation; IQR, interquartile range. ^a^*P *< 0.05.

#### Signs

On admission to the ICU, heart rates and respiratory rates were similar for IBPA and non-IBPA patients. Mean arterial blood pressures were significantly lower for IBPA patients than for non-IBPA patients (78 mm Hg versus 91 mm Hg; *P *= 0.019). Dry rales were heard more frequently in the lungs of IBPA patients (85% versus 40%; *P *= 0.005) (Table 3).

### Multivariate analysis

Variables with a *P *value < 0.1 in the univariate analysis are shown in Tables 1 and 3. Of these, three were included in the multivariate model: more than three kinds of antibiotics used before the ICU admission, accumulated doses of corticosteroids (>350 mg) received before the ICU admission, and APACHE II scores >18. The multivariate analysis selected the three variables with independent statistical significance (Table 4).

**Table 4:** Variables selected for prediction of invasive bronchopulmonary aspergillosis by multivariate logistic regression analysis in patients with chronic obstructive respiratory disease

	Wald	*P *Value	Odds ratio	95% Confidence interval	
				
				Inferior	Superior
Accumulated dosage of systemic steroids (>350 mg) received before the ICU admission	4.326	0.038	8.661	1.133	66.239
More than three kinds of antibiotics before the ICU admission	5.211	0.022	1.208	1.027	1.422
APACHE II scores >18	6.974	0.008	19.488	2.150	176.613
Constant	11.912	0.001			

APACHE, Acute Physiology and Chronic Health Evaluation; ICU, intensive care unit.

### Laboratory tests

White blood cell (WBC) counts were significantly higher for IBPA patients on days 1, 4, and 7 of ICU admission. The pH and base excess (BE) were significantly lower for IBPA patients on the first day, but were not different on days 4 and 7. Serum creatinine clearances were significantly decreased for IBPA compared with non-IBPA patients on days 1, 4, and 7 of ICU admission. During their ICU stays, IBPA patients had significantly higher serum aspartate aminotransferase levels, alanine aminotransferase levels, and activated partial thromboplastin times (see Table 5, Figure S1 in Additional file 1, and Figure S2 in Additional file 2).

**Table 5:** Laboratory findings

	Day of ICU admission	IBPA	Control	*P *value
Complete blood count				
White blood cell count (×10^9^/L), mean (SD)	Day 1	21.0(14.0)	9.4(3.7)	0.012^a^
	Day 4	17.5(5.6)	10.6(13.9)	0.101
	Day 7	19.5(6.3)	10.0(5.4)	0.000^a^
Neutrophilic granulocyte (%), mean (SD)	Day 1	90.2(6.3)	84.3(10.0)	0.053
	Day 4	89.4(7.7)	79.3(10.7)	0.004^a^
	Day 7	87.8(6.9)	80.3(8.6)	0.027^a^
Arterial blood gas analysis				
pH, mean (SD)	Day 1	7.25(0.14)	7.36(0.11)	0.005^a^
	Day 4	7.42(0.08)	7.42(0.05)	0.768
	Day 7	7.42(0.10)	7.42(0.05)	0.995
PaCO_2 _(mm Hg), mean (SD)	Day 1	65.3(36.1)	68.8(33.1)	0.745
	Day 4	53.3(22.7)	53.5(11.4)	0.979
	Day 7	47.9(22.4)	49.2(12.9)	0.845
Ratio of the PaO_2 _to FiO_2_, mean (SD)	Day 1	166.0(86.8)	219.1(128.8)	0.171
	Day 4	197.5(80.0)	225.7(88.2)	0.332
	Day 7	199.9(72.3)	236.8(83.8)	0.255
Renal function				
Clearance of creatinine (ml/min), mean (SD)	Day 1	36.2(20.4)	68.8(27.5)	0.000^a^
	Day 4	36.6(24.0)	82.5(51.5)	0.005^a^
	Day 7	33.3(32.3)	77.6(50.4)	0.021^a^
Liver function				
ALT (U/L), median (IQR)		52.5(36.5-95)	28(20-43)	0.003^a^
AST (U/L), median (IQR)		67(49-118.5)	26.5(21.8-49.8)	0.000^a^
Coagulation				
APTT (s), median (IQR)		34.8(28.4-49.3)	28.5(26.2-37.7)	0.046^a^

ALT, alanine aminotransferase; APTT, activated partial thromboplastin time; AST, aspartate aminotransferase; FiO_2_, the fraction of inspired oxygen; SD, standard deviation; IQR, interquartile range; PaCO_2_, partial pressure of arterial carbon dioxide; PaO_2_, partial pressure of arterial oxygen. ^a^*P *< 0.05.

### Fiberoptic bronchoscopy

On days 1, 4, and 7 after ICU admission, 11, 10, and four IBPA patients and 15, six, and three non-IBPA patients had bronchoscope examinations, respectively. For IBPA patients, mucous hyperemia and edema were observed, ropy sputum was difficult to suck out, and four cases showed pseudomembrane formation under bronchoscopic analysis. Bronchospasm, plug formation and sputum ropiness were more common for IBPA on the first day after ICU admission (66.7% versus 14.3%; *P *= 0.082; 18% versus 0; *P *= 0.169; and 73% versus 13%; *P *= 0.003, respectively). Four patients in the IBPA group had biopsies of the tracheobronchial tree during bronchoscopy, which showed *Aspergillus *invasion into the tracheobronchial wall.

### Radiologic examination

On days 1, 4, and 7 after ICU admission, 13, 12, and nine IBPA patients and 42, 32, and 22 non-IBPA patients had radiologic examinations, respectively. In each group, six patients had chest CTs on the first day of ICU admission. Among the six cases with IBPA, one had a halo sign, and one had a cavity on the CT scans. The CT scans of the other four IBPA cases and the six non-IBPA patients showed nonspecific patching, nodules, and consolidations. The numbers of IBPA patients with nodules and consolidations on CXR increased rapidly from day 1 to day 7 of ICU admission (nodules: from three patients to six patients; consolidations: from one patient to five patients). Compared with non-IBPA patients, patchiness and nodules were more common on CXR on day 1 of admission for IBPA patients (77% versus 43%, *P *= 0.032; and 23% versus 2.3%, *P *= 0.012, respectively). At day 4, no significant differences were found between the two groups, and at day 7, nodules and consolidations were significantly more common for the IBPA patients (60% versus 9%, *P *= 0.002; and 50% versus 14%, *P *= 0.028) (see Figure S3 in Additional file 3).

### Serum GM test

The sensitivities, specificities, positive and negative predictive values, and total consistent rates for positive GM results of a first test and of a second test, at least one positive GM result from two consecutive tests, and both positive GM results of two consecutive tests are shown in Table 6. The total consistent rates did not show significant differences between different diagnostic strategies.

**Table 6:** Results of first and two consecutive detections of galactomannan in serum of critically ill CORD patients

	Single GM detection (95% CI)		Two consecutive GM detections (95% CI) (*n *= 48)	
		
	Positive for a first test (*n *= 55)	Positive for a second test (*n *= 48)	At least one positive of the two consecutive tests	Both positive for the two consecutive tests
Sensitivity (%)	46.2 (33.3-59.1)	50.0 (35.9-54.1)	53.8 (39.7-67.9)	41.7 (27.8-55.6)
Specificity (%)	83.3 (73.4-93.2)	93.5 (86.5-100)	81.0 (69.9-92.1)	93.5 (86.5-100)
PPV (%)	46.2 (33.3-59.1)	75.0 (62.7-87.3)	46.7 (32.6-60.8)	71.4 (58.7-84.1)
NPV (%)	83.3 (73.4-93.2)	82.9 (72.2-93.6)	85.0 (74.9-95.1)	80.6 (69.7-91.5)
TCR (%)	74.5 (63.0-86.0)	81.4 (70.4-91.4)	74.5 (62.2-86.8)	79.1 (67.9-90.3)

CORD, chronic obstructive respiratory disease; NPV, negative predictive value; PPV, positive predictive value; TCR, total consistent rate.

### Diagnostic algorithm

**Figure 1:**
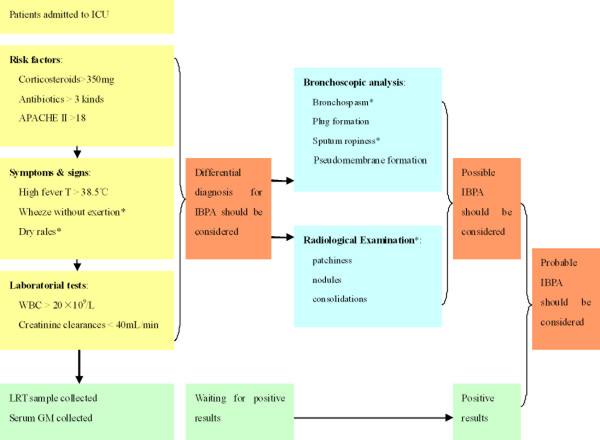
**Diagnostic algorithm based on patient's clinical features and diagnostic procedures for IBPA in critically ill CORD patients admitted in ICU**. *Resistant to appropriate treatment including corticosteroids and antibiotics.

Based on the risk factors, symptoms and signs, and diagnostic procedures evaluated in our study, a diagnostic algorithm is shown in Figure 1.

## Discussion

The main strength of our study is its prospective design that included non-IBPA CORD patients as a control group. This enabled us to discriminate between IBPA in the CORD population and acute exacerbations caused by CORD itself. Our study revealed that before ICU admission, three variables were independent predictors of IBPA: more than three kinds of antibiotics used before the ICU admission, accumulated doses of corticosteroids (>350 mg) received before the ICU admission, and APACHE II scores >18. In critically ill CORD patients, IBPA may present as respiratory failure and clinical and bronchoscopic manifestations of severe infection, bronchospasm, and rapid progression of radiologic lesions that are unresponsive to steroids and antibiotics.

**Figure 2:**
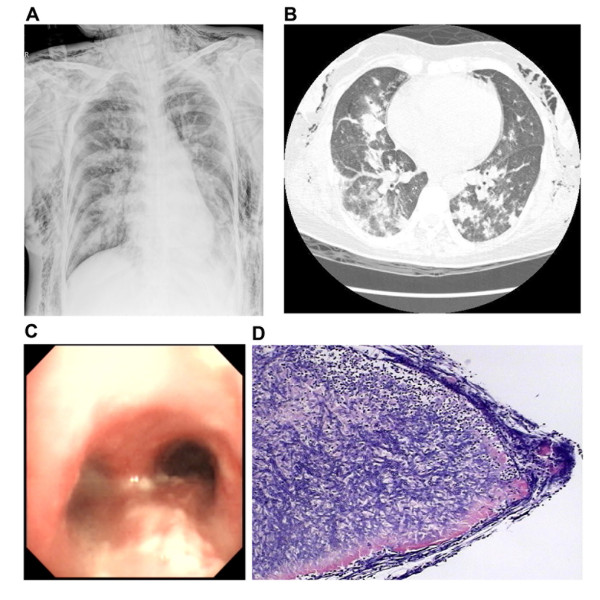
**Radiologic, bronchoscopic, and histologic information of Patient IBPA Case 2**. **(a) **Chest x-ray shows patchings and multiple nodules in bilateral lungs, with severe barotrauma. **(b) **Chest HRCT shows patchings and multiple nodules with halo sign in bilateral lungs. **(c) **Bronchoscopy shows inflammation and white plaques formation of the airway. **(d) **Histologic examination of biopsy specimen from lung tissue shows many *Aspergillus *hyphae invading the lung parenchyma.

In CORD patients, because the structures and defense functions of the airways and lung parenchyma are damaged by their underlying respiratory diseases, *Aspergillus *may colonize in these sites. During the early period of invasive aspergillosis, infection may be limited to the tracheobronchial tree, presenting as ATB. This may account for the obvious bronchospasm without radiologic appearance in some cases during the early phase of infection. With corticosteroids and broad-spectrum antibiotics therapy, the infection could spread to the distal airways and lung parenchyma, presenting as IPA. Several reports have shown that lung parenchyma was usually involved together with ATB, and invasive ATB may indicate an advanced pulmonary lesion caused by *Aspergillus *[11-15]. In our study, two patients who had a tracheobronchial mucus and lung biopsy (cases 2 and 12) had specific radiologic findings on their CT scans, as well as positive GM tests. In addition, in case 2, the lesions of the airways and lung parenchyma responded to antifungal treatment, which suggested a concomitant pulmonary lesion secondary to *Aspergillus *(see Figure 2). Therefore, ATB may be an early stage of IPA, and may exist either before or with IPA.

For CORD patients, corticosteroids treatment is considered to be an important risk factor for IBPA [4,7,10,16]. The conicidal activity of human tissue macrophages is responsible for the monocyte-mediated damage to fungal hyphae [17], and this immune function could be impaired by corticosteroids [18]. Moreover, corticosteroids promote the *in vitro *growth of *Aspergillus fumigatus *[19]. Previous studies reported that COPD patients who were given an average daily dose of systemic corticosteroids greater than 73 to 80 mg of prednisone (or equivalent), and with an average therapy duration of 29.7 days to 2 months were prone to developing invasive aspergillosis [6,8,20]. However, another retrospective study showed that an accumulated dosage of steroids equivalent to >700 mg of prednisone received during the 3 months before hospital admission may be a risk factor for IPA in COPD patients [21]. In our study, the patients in the IBPA and non-IBPA groups received the same daily dosages of steroids. However, because the IBPA patients received them for a longer period (median, 6 days versus 1 day), the accumulated steroid dosages in this group may have been higher than those in the non-IBPA group. According to the multivariate analysis in our study, an accumulated dosage of 350 mg prednisone may be associated with IBPA in critically ill CORD patients. For the two IBPA patients who had positive microbiologic results for LRT samples collected at days 6 and 8 after the ICU admission, no corticosteroids were used after their ICU admission, which means that for the 13 IBPA cases, the "accumulated dosage before the admission to the ICU" was the same as "accumulated dose of corticosteroids before the first isolation of *Aspergillus*" proposed in a previous study [21].

Antibiotic therapy before admission to an ICU could also be an important risk factor. This was observed, but not confirmed, in two retrospective studies [8,22]. Muquim *et al. *[20] reported that IPA that occurred with COPD was associated with the use of multiple broad-spectrum antibiotics before patients' hospitalizations, and the risk for IPA increased with the number of antibiotics used. The number of antibiotics used may suggest a pneumonia that did not respond to several antibiotic treatments. In our study, a median of three or more antibiotics for 10 days may have been a risk factor for IBPA for the critically ill CORD patients before their ICU admissions.

CORD patients are not immunocompromised, although some of them may be mildly immunosuppressed. Therefore, when their airways or lung parenchyma are invaded by *Aspergillus*, their immune systems should react to this pathogen, and they may show a severe systemic inflammatory reaction [7] and obvious bronchospasm [23]. High fever (T >38.5°C) and elevated WBC (>20 × 10^9^/L) are systemic manifestations of inflammatory reactions. The trend of low arterial pressure, acidosis, acute renal dysfunction (creatinine clearance <36 ml/min), and abnormalities of liver function and coagulation in critically ill CORD patients may suggest the presence of a more severe inflammatory status, such as septic shock and multiorgan dysfunction, which are the main causes of death for IBPA patients. Dyspnea without exertion and diffuse wheezing rales in the lungs are manifestations of severe bronchospasm, which may suggest a local inflammatory status of the airways. The presentation of severe inflammatory status and bronchospasm are not specific for IBPA; however, when they are resistant to appropriate antibiotics and corticosteroids, a diagnosis of IBPA should be suspected.

A local inflammatory reaction can also be observed directly during bronchoscopy, presenting as mucous hyperemia, edema, large amounts of ropy airway secretions, plaque, pseudomembrane formation, and bronchospasm. Further, performing bronchoscopy with microscopic examinations of tracheal or bronchial specimens is the most sensitive diagnostic test for an early diagnosis and treatment of ATB [2]. In our study, four patients had ATB presentations during bronchoscopic analysis, and all of them were confirmed as proven ATB with biopsies of tracheobronchial tissues. A previous study suggested that when IPA is suspected, but culture evaluations of nonbronchoscopic samples alone are negative, intubation and bronchoscopy should be considered [8]. Therefore, bronchoscopy is necessary for establishing an early diagnosis of ATB.

Risks exist for ICU patients in having chest CTs because of their severe conditions and difficulties with transportation. As a result, radiologic data for this population is mainly dependent on bedside CXR. However, unlike immunocompromised patients, early findings on CXR or CT scan for IBPA in CORD patients are nonspecific, and halo signs and cavitations are uncommon [4,16,20,24]. Moreover, for some ATB patients, no obvious abnormalities could be found on their chest images. Therefore, it is difficult to establish an early diagnosis of IBPA based on classic manifestations on their chest CTs or CXRs for CORD patients. Further, our study suggested the rapid progression of patching to nodules and consolidations in multiple segments and lobes, which were unresponsive to empiric antibacterial agents. Therefore, the rapid progression on a chest image may be suggestive of suspected IBPA.

In our study, a high proportion of IBPA patients had a positive culture for *Aspergillus *in the same day (day 1) of ICU admission (84.6%, 11 of 13). In the 11 cases, no patients had the diagnosis of IA before their admission, no *Aspergillus *was isolated previous to the ICU admission, and none of these patients received antifungal agents before ICU admission. It means that, probably, these patients were admitted to the unit because of the IA, which can partly explain why the mortality was as high as 69% in our study.

We realize that this study has limitations. First, to perform multivariate analysis on a small dataset (three predictor variables for 13 IBPA cases) was prone to bias and model overfitting, yielding spurious findings, which made a large 95% CI for OR in our analysis. Increasing the sample size and collecting more IBPA cases in a further study may avoid this kind of limitation. Second, only four patients had biopsies and were diagnosed as having proven IBPA, which may cause possible misclassification bias. Finally, this is a single-center study, in which a setting may have tremendous overuse of antibiotic agents for empiric treatment and prophylaxis. A multicenter prospective study maybe needed in the future to avoid this kind of limitation.

## Conclusions

In conclusion, IBPA is not rare among critically ill CORD patients with ICU admissions, with mortality as high as 69%. IBPA may be suspected in critically ill CORD patients with respiratory failure and clinical and bronchoscopic manifestations of severe infection (high fever, elevated WBC count, low blood pressure and multiorgan dysfunction, sputum ropiness, and plaque formation), bronchospasm (wheeze and dry rales), and rapid progression of radiologic lesions, which are irresponsive to steroids and antibiotics. To avoid misdiagnosis and to establish the microbiologic etiology, early bronchoscopy and tight radiologic follow-up should be performed.

## Key messages

• ATB may be an early stage of IPA and may exist either before or with IPA. Therefore, ATB and IPA might be two phases or manifestations of one entity, invasive bronchopulmonary aspergillosis (IBPA).

• In critically ill CORD patients, before ICU admission, three variables were independent predictors of IBPA: more than three kinds of antibiotics used before the ICU admission, accumulated doses of corticosteroids (>350 mg) received before the ICU admission, and APACHE II scores >18.

• In critically ill CORD patients, IBPA may present as respiratory failure and clinical and bronchoscopic manifestations of severe infection, bronchospasm, and rapid progression of radiologic lesions that are irresponsive to steroids and antibiotics.

• IBPA is not rare among critically ill CORD patients with ICU admissions, with mortality as high as 69%.

## Abbreviations

ABGA: arterial blood gas analysis; APACHE II: the Acute Physiology and Chronic Health Evaluation II; APTT: activated partial thromboplastin time; ATB: *Aspergillus *tracheobronchitis; BE: base excess; COPD: chronic obstructive pulmonary disease; CORD: chronic obstructive respiratory disease; CT: computed tomography; CXR: chest x-ray; EORTC/MSG: the European Organization for Research and Treatment of Cancer/Mycoses Study Group; GM: galactomannan; IA: invasive aspergillosis; IBPA: invasive bronchial-pulmonary aspergillosis; ICU: intensive care unit; IPA: invasive pulmonary aspergillosis; LRT: lower respiratory tract; WBC: white blood cell.

## Competing interests

The authors declare that they have no competing interests.

## Authors' contributions

All authors made substantial contributions to conception and design, or acquisition of data, or analysis and interpretation of data; reviewed and approved the final manuscript; and contributed significantly to this study. Drs HH and LD contributed equally to the work. QZ takes full responsibility for the integrity of the submission and publication and was involved in study design. HH had full access to all the data in the study, takes responsibility for the integrity of the data and the accuracy of the data analysis, and was responsible for the data verification, analysis, and drafting of the manuscript. LD had full access to all the data in the study and takes responsibility for the integrity of the data and the accuracy of the data analysis. FL was responsible for the microbiologic examination and the data collection.

## Supplementary Material

Additional file 1 **Figure S1**. Arterial blood gas analysis and blood cell count after RICU admission. Click here for file

Additional file 2 **Figure S2**. Biochemical and coagulation test after RICU admission. Click here for file

Additional file 3 **Figure S3**. Comparison of the chest radiologic presentation between the two groups. Click here for file
