# Supplementary material for: Clinical features of invasive bronchial-pulmonary aspergillosis in critically ill patients with chronic obstructive respiratory diseases: a prospective study
Source: Crit Care. 2011 Jan 6;15(1):R5. doi: 10.1186/cc9402 (PMC3222032; doi:10.1186/cc9402)
Supplement: Additional file 1 — Figure S1. Arterial blood gas analysis and blood cell count after RICU admission. [file cc9402-S1.PDF]

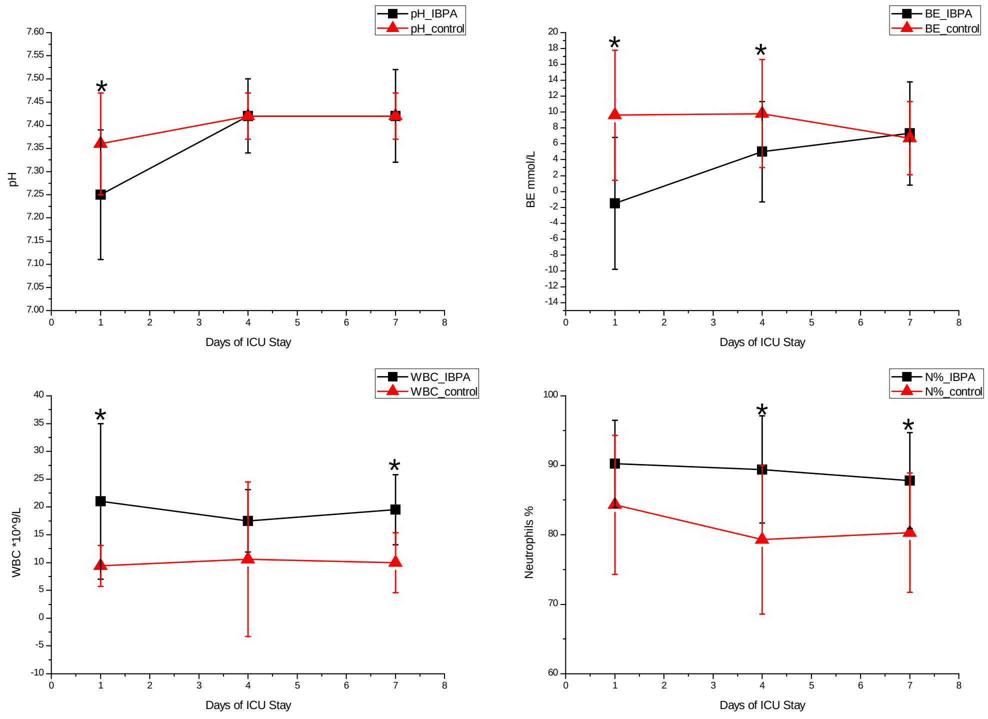

Figure S1. Arterial blood gas analysis and blood cell count after RICU admission.

BE = base excess; WBC = white blood cell; Presented as means ± SD. ⋆P<0.05
