# Supplementary material for: Clinical features of invasive bronchial-pulmonary aspergillosis in critically ill patients with chronic obstructive respiratory diseases: a prospective study
Source: Crit Care. 2011 Jan 6;15(1):R5. doi: 10.1186/cc9402 (PMC3222032; doi:10.1186/cc9402)
Supplement: Additional file 2 — Figure S2. Biochemical and coagulation test after RICU admission. [file cc9402-S2.PDF]

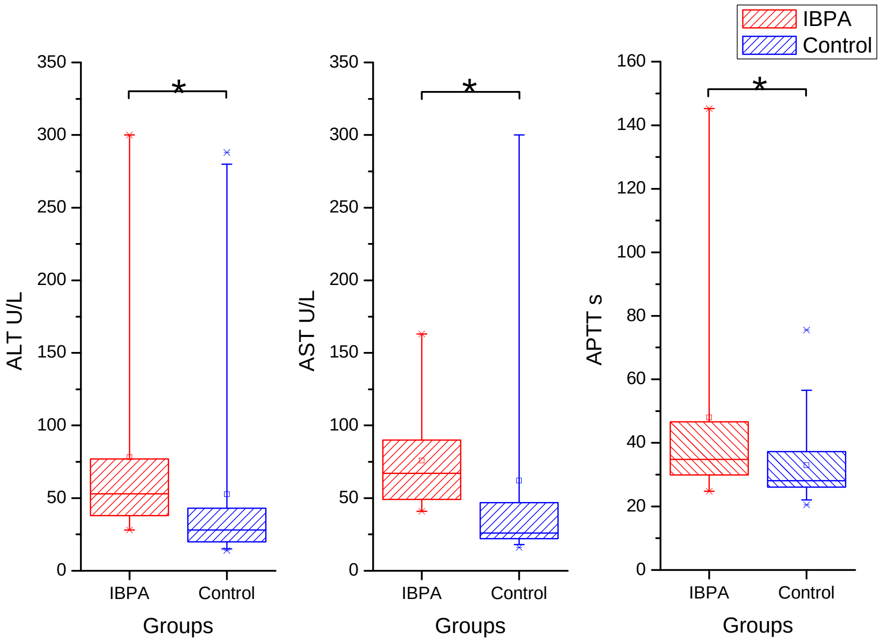

Figure S2. Biochemical and coagulation test after RICU admission.

ALT = alanine aminotransferase; AST = aspartate aminotransferase; APTT = activated partial thromboplastin time. Presented as 5 and 95 percentile (whisker) and interquartile (box). * $P<0.05$
