# Supplementary material for: Clinical features of invasive bronchial-pulmonary aspergillosis in critically ill patients with chronic obstructive respiratory diseases: a prospective study
Source: Crit Care. 2011 Jan 6;15(1):R5. doi: 10.1186/cc9402 (PMC3222032; doi:10.1186/cc9402)
Supplement: Additional file 3 — Figure S3. Comparison of the chest radiologic presentation between the two groups. [file cc9402-S3.PDF]

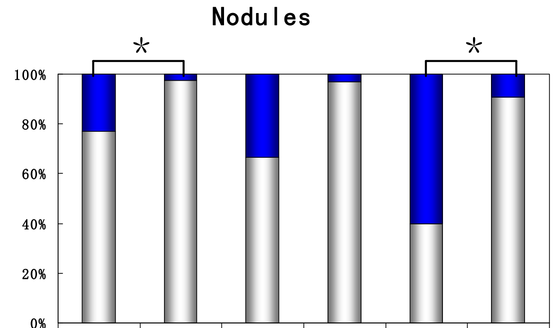

	IBPA d1	Control d1	IBPA d4	control d4	IBPA d7	Control d7
Positive	3	1	4	1	6	2
Negtive	10	41	8	31	4	20

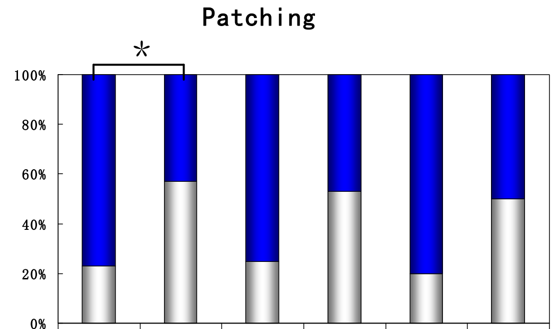

	IBPA d1	Control d1	IBPA d4	control d4	IBPA d7	Control d7
Positive	10	18	9	15	8	11
Negtive	3	24	3	17	2	11

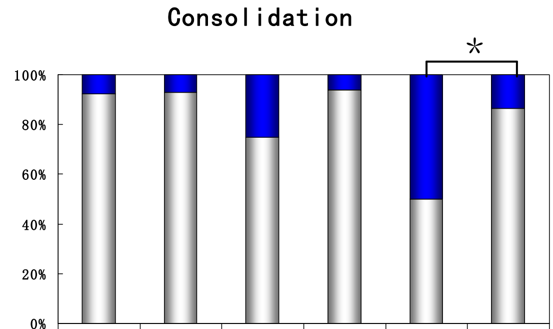

	IBPA d1	Control d1	IBPA d4	control d4	IBPA d7	Control d7
Positive	1	3	3	2	5	3
Negtive	12	39	9	30	5	19

Figure S3. Comparison of the chest radiological presentation between the two groups.

The number of patients take radiological examination on day 1, 4 and 7 are list under the figures respectively. ＊P<0.05
